# Genetic Characterization of a Conjugative Plasmid That Encodes Azithromycin Resistance in *Enterobacteriaceae*

**DOI:** 10.1128/spectrum.00788-22

**Published:** 2022-04-26

**Authors:** Xiaoxuan Liu, Xuemei Yang, Lianwei Ye, Edward Wai-Chi Chan, Sheng Chen

**Affiliations:** a Department of Infectious Diseases and Public Health, Jockey Club College of Veterinary Medicine and Life Sciences, City University of Hong Kong, Kowloon, Hong Kong; b State Key Lab of Chemical Biology and Drug Discovery, Department of Applied Biology and Chemical Technology, The Hong Kong Polytechnic University, Hung Hom, Hong Kong; University of Greifswald

**Keywords:** *Klebsiella pneumoniae*, *Salmonella*, azithromycin resistance, *erm*(B), *erm*(42), *mph*(A)

## Abstract

Mechanisms of azithromycin resistance have rarely been reported. In this study, an IncFIB/IncHI1B plasmid that confers resistance to azithromycin was recovered from a clinical Klebsiella pneumoniae strain. This plasmid could be efficiently disseminated to Escherichia coli, Salmonella, and other Gram-negative bacterial pathogens through conjugation. This plasmid was shown to carry three macrolide resistance genes: *erm*(B), a novel *erm*(42) gene, and *mph*(A). The functions of *erm*(42) were confirmed by direct cloning of this gene and determination of the MIC of azithromycin in strains of various bacterial species which have acquired this gene. Of particular concern is the potential transmission of azithromycin-resistance to extensively drug-resistant (XDR) Salmonella, which causes infections for which treatment options are extremely limited. Monitoring and preventing dissemination of this azithromycin resistance-encoding conjugative plasmid in *Enterobacteriaceae* is of utmost importance.

**IMPORTANCE** In this study, we identified a conjugative plasmid carrying a novel azithromycin resistance gene, *erm*(42), from a clinical K. pneumoniae strain. Conjugation of this plasmid into Salmonella conjugants conferred resistance to azithromycin, which is considered a choice for treating Salmonella infections. Of particular concern is the dissemination of this type of azithromycin resistance-encoding conjugative plasmid to extensively drug-resistant (XDR) Salmonella. The study shows that further monitoring of the dissemination of this plasmid in clinical strains of Salmonella spp. is warranted.

## INTRODUCTION

Macrolides are a family of oral antibiotics that consist of a large macrocyclic lactone ring to which amino sugars and neutral sugars are attached (1). Clinical use of macrolides has steadily increased since the introduction of erythromycin into clinical practice in 1952 (2). Erythromycin exerts its antimicrobial activity by disrupting the function of the ribosome to block protein synthesis (3, 4). It has long been regarded as a substitute for penicillin that can be used for treatment of respiratory, skin, and soft tissue infections, with a wide spectrum of activity and good safety record (5). However, the potential of erythromycin as a general-use oral antibiotic is limited by its acidic stability and oral bioavailability. To improve its biological profile, modification of the basal structure of macrolides resulted in the formation of new subgroups of macrolides, such as azalides, of which azithromycin has become the first and most representative member (1). The advantages of azithromycin in clinical treatment are its high tissue distribution, metabolic stability, and high tolerability (1). Although macrolides have generally not been chosen for treatment of *Enterobacteriaceae* infections due to their relatively low antimicrobial activity (6, 7), some macrolides, such as azithromycin, possess excellent clinical parameters against *Enterobacteriaceae* (8, 9). With a methyl-substituted nitrogen in the macrolide ring that differs structurally from erythromycin, azithromycin exhibits higher permeation of the outer membrane of *Enterobacteriaceae* due to its hydrophobic nature (10).

Bacterial mechanisms that confer resistance to macrolides include (i) target site alteration, (ii) antibiotic modification, and (iii) altered antibiotic transport (11). Macrolide resistance was first attributed to methylation of 23S rRNA by a class of genes, the *erm* (erythromycin ribosome methylation) genes, which encode a group of structurally homologous methylases that modify a single adenine residue in the 23S rRNA to form either *N*^6^-mono- or dimethyladenine (11), inhibiting interaction between the macrolide molecule and the ribosome. In this study, we identified a multidrug resistance plasmid that harbors two erythromycin ribosome methylase genes, *erm*(B) and *erm*(42). Recovered from a clinical Klebsiella pneumoniae strain, this plasmid was found to be conjugative and able to confer resistance to azithromycin in a variety of *Enterobacteriaceae* strains. Recently, our laboratory reported the identification of a plasmid-borne *erm*(B) gene in a clinical K. pneumoniae strain which mediated azithromycin resistance in different bacterial species (12). At least six different transferable *erm* genes—*erm*(B), *erm*(C), *erm*(D), *erm*(E), *erm*(F), and *erm*(42)—have currently been described in *Enterobacteriaceae* (13). However, the role of *erm*(42) in mediating azithromycin resistance has not been elucidated. The *erm*(42) gene was first reported in the animal pathogens Mannheimia haemolytica and Pasteurella multocida in 2011 (14) and then became detectable in a type 2 A/C plasmid from Salmonella enterica in 2015 (15). It is phylogenetically distant from the other *erm* family members like *erm*(A) and *erm*(B), with the closest known orthologue being *erm*(Q) (39% identity in the core sequence) (14). Nevertheless, the *erm*(42) gene is known to confer resistance to macrolides such as erythromycin, tilmicosin, and clindamycin (16). Here, we describe the functional role of *erm*(42) in mediating resistance to azithromycin in different *Enterobacteriaceae* strains.

## RESULTS AND DISCUSSION

K. pneumoniae strain EH13, which was confirmed by matrix-assisted laser desorption ionization–time of flight (MALDI-TOF), was recovered from a patient in a hospital in Hong Kong SAR in 2017. Antimicrobial susceptibility testing showed that EH13 was resistant to azithromycin, the β-lactam antibiotics ampicillin, aztreonam, cefotaxime, and ceftazidime, and the aminoglycoside amikacin, as well as ciprofloxacin and chloramphenicol, but remained susceptible to gentamicin, meropenem, colistin, and tigecycline (Table 1).

**TABLE 1:** Phenotypic and genotypic characteristics of K. pneumoniae strain EH13 and its transconjugants

Strain	Organism	MIC (μg/mL)^*a*^												*erm*(42)^*b*^
		AZI	CTX	CAZ	CIP	CHL	ATM	AMP	AMK	GEN	MEM	CLS	TGC	
EH13	K. pneumoniae	256	>128	>128	8	32	128	>128	4	0.5	0.25	2	2	+
J53	E. coli	0.25	0.125	0.125	0.125	2	0.125	4	2	0.5	0.125	2	0.25	−
J53TC	E. coli	128	128	1	0.125	8	2	>128	2	1	0.125	2	0.25	+
PY1	S. enterica subsp*. enterica* serovar Typhimurium	4	0.125	0.25	0.125	8	2	2	2	1	0.125	2	0.5	−
PY1TC	S. enterica subsp*. enterica* serovar Typhimurium	512	>128	4	0.125	8	16	>128	2	1	0.125	2	0.5	+
25922	E. coli	2	<0.25	0.5	0.125	4	<0.25	8	4	4	0.125	0.5	0.25	NA

a All tests were performed in duplicate, and each test included three biological replicates. AZI, azithromycin; CTX, cefotaxime; CAZ, ceftazidime; CIP, ciprofloxacin; CHL, chloramphenicol; ATM, aztreonam; AMP, ampicillin; AMK, amikacin; GEN, gentamicin; MEM, meropenem; CLS, colistin; TGC, tigecycline.

b +, *erm*(42) gene positive; −, *erm*(42) gene negative; NA, not available.

**FIG 1:**
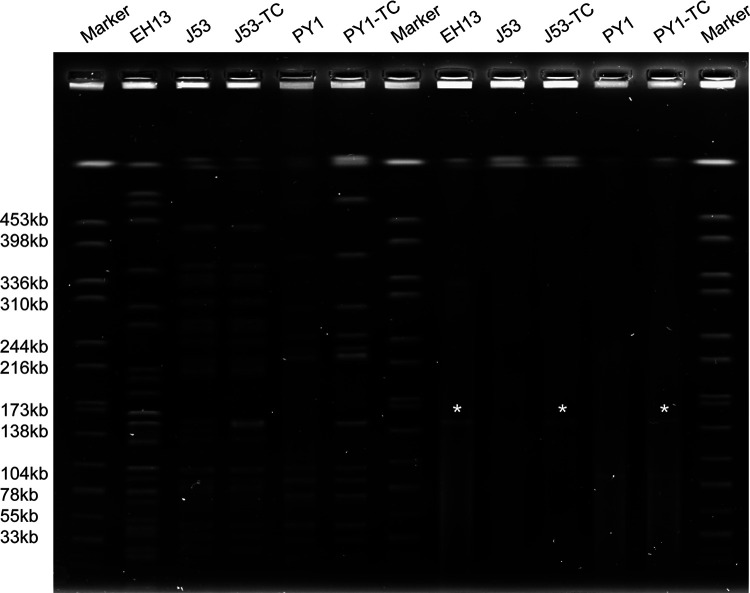
XbaI-PFGE and S1-PFGE analysis of strain EH13, recipient strain E. coli J53, *S.* Typhimurium PY1, and their corresponding transconjugants, J53TC and PY1TC. Stars indicate the conjugative plasmid pEH13_2. XbaI- and S1-PFGE were repeated twice for all test strains, and identical results were obtained.

The complete genome of strain EH13 was obtained and shown to include a chromosome of 5,301,226 bp and two plasmids of 330,084 bp and 152,520 bp, which was consistent with the results of S1 nuclease pulsed-field gel electrophoresis (S1-PFGE) (Fig. 1). Strain EH13 was found to belong to ST70-1LV and KL122 by Kleborate. This strain was found to carry a wide range of resistance genes, including the quinolone resistance genes *oqxAB*, the beta-lactam resistance genes *bla*_DHA-1_, *bla*_TEM-1B_, *bla*_CTX-M-14_, and *bla*_SHV-32_, the aminoglycoside resistance genes *aph(6)-Id*, *aadA16*, *ant(3″)* and *ant(3″)-Ia*, the macrolide resistance genes *mph*(A), *erm*(B) and *erm*(42), the fosfomycin resistance gene *fosA*, the sulfonamide resistance genes *sul1* and *sul2*, the tetracycline resistance genes *tet*(M) and *tet*(D), and the chloramphenicol resistance gene *cmlA1*. Except for the *bla*_SHV-32_, *fosA*, and *oqxAB* genes, the resistance genes were plasmid borne.

**FIG 2:**
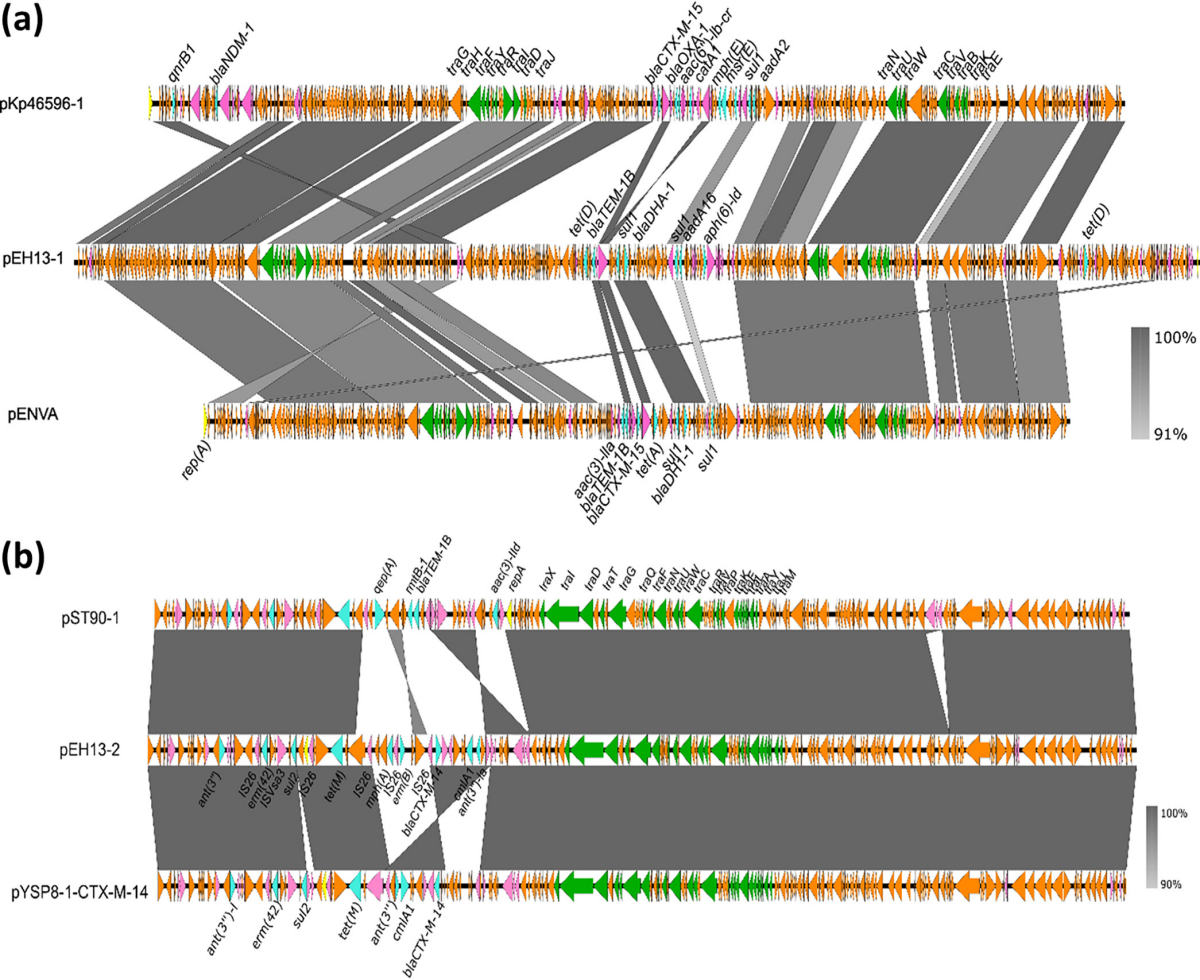
Alignment of pEH13_1 (a) and pEH13_2 (b) with structurally similar plasmids using Easyfig.

The *tet*(D), *aph(6)-Id*, *aadA16*, *sul1*, *bla*_DHA-1_, and *bla*_TEM-1B_ genes were found to be located on a 330,084-bp plasmid designated pEH13_1 (Fig. 2a). Plasmid pEH13_1 was an IncFIB/IncHI1B hybrid plasmid that possesses 381 coding sequences, with a GC content of 47.5%. BLAST results showed that it exhibited the highest degree of sequence similarity (76% coverage and 97.79% identity) to the 253,984-bp plasmid pENVA (GenBank accession no. HG918041.1) and the 286,241-bp plasmid pKp46596-1 (GenBank accession no. CP050311.1; 69% coverage and 99.98% identity), both recovered from K. pneumoniae strains. The *ant(3″)*, *erm*(42), *sul2*, *tet*(M), *mph*(A), *erm*(B), *bla_CTX-M-14_*, *cmlA1*, and *ant(3″)-Ia* genes were found to be located on a 152,520-bp plasmid designated pEH13_2 (Fig. 2b). Plasmid pEH13_2 contains two replicon loci, IncFII and IncFIA, and possesses 173 coding sequences, with a GC content of 51.1%. It exhibited the highest degree of sequence similarity to the 149,304-bp plasmid pYSP8-1-CTX-M-14 (GenBank accession no. CP037912.1; 97% coverage and 99.80% identity) and the 150,326-bp plasmid pST90-1 (GenBank accession no. CP050735.1; 90% coverage and 99.98% identity), which were recovered from E. coli and K. pneumoniae strains, respectively. pYSP8-1-CTX-M-14 harbors the same resistance genes as in pEH13_2, except for *mph*(A) and *erm*(B); pST90-1 also possesses the region containing the resistance genes *ant(3″)*, *erm*(42), *sul2*, *tet*(M), *bla_CTX-M-14_*, *cmlA1*, and *ant(3″)-Ia*, which can be found in pEH13_2 (Fig. 2b). The pEH13_2 plasmid also carries the *iuc* locus, which encodes the virulence factor aerobactin. Aerobactin is a hypervirulent K. pneumoniae (hvKp)-specific siderophore that accounts for >90% of the siderophore activity, despite the fact that hvKP produces multiple siderophores (17). Aerobactin is the primary virulence determinant among hvKp’s siderophores and is responsible for causing systemic infection (18). It is also a critical cellular factor requisite for optimal growth in human ascites fluid *ex vivo* and plays an important role in growth and survival outside of the living body (17, 19).

The *erm*(B), *erm*(42), and *mph*(A) genes all encode macrolide resistance, among which *erm*(B) and *erm*(42) belong to the *erm* (erythromycin ribosome methylation) family. *erm*(B) has been shown to mediate azithromycin resistance, but direct evidence which shows that *mph*(A) and *erm*(42) can mediate azithromycin resistance is not available (12). To elucidate the role of *erm*(42) in encoding azithromycin resistance, the gene and its regulatory region in plasmid pEH13_2 were cloned and introduced into Escherichia coli strain DH5a and S. enterica subsp. *enterica* serovar Typhimurium strain PY1. The MICs of azithromycin for strains DH5α and PY1 were 2 and 4 μg/mL, respectively, while DH5α and PY1 strains which had acquired the *erm*(42) gene through transformation were able to encode high-level resistance to azithromycin (both 64 μg/mL), indicating that *erm*(42) can mediate resistance to azithromycin.

The transferability of plasmid pEH13_2 was determined by conjugation using E. coli strain J53 as the recipient. The results showed that pEH13_2 could be directly transferred from K. pneumoniae EH13 to E. coli J53. XbaI- and S1-PFGE confirmed the acquisition of a 150-kb plasmid in J53 transconjugants (Fig. 1). Transferability of plasmid pEH13_2 to Salmonella was further detected using S. enterica strain PY1 as the recipient. The plasmid pEH13_2 was able to be transferred to strain PY1 from strain J53 according to the PFGE result. Furthermore, acquisition of pEH13_2 rendered the transconjugants resistant to azithromycin, as well as cefotaxime and ampicillin (Table 1). It is worth noting that pEH13_2 was able to mediate an even higher level of resistance to azithromycin in PY1 than in its original host EH13 (Table 1).

**FIG 3:**
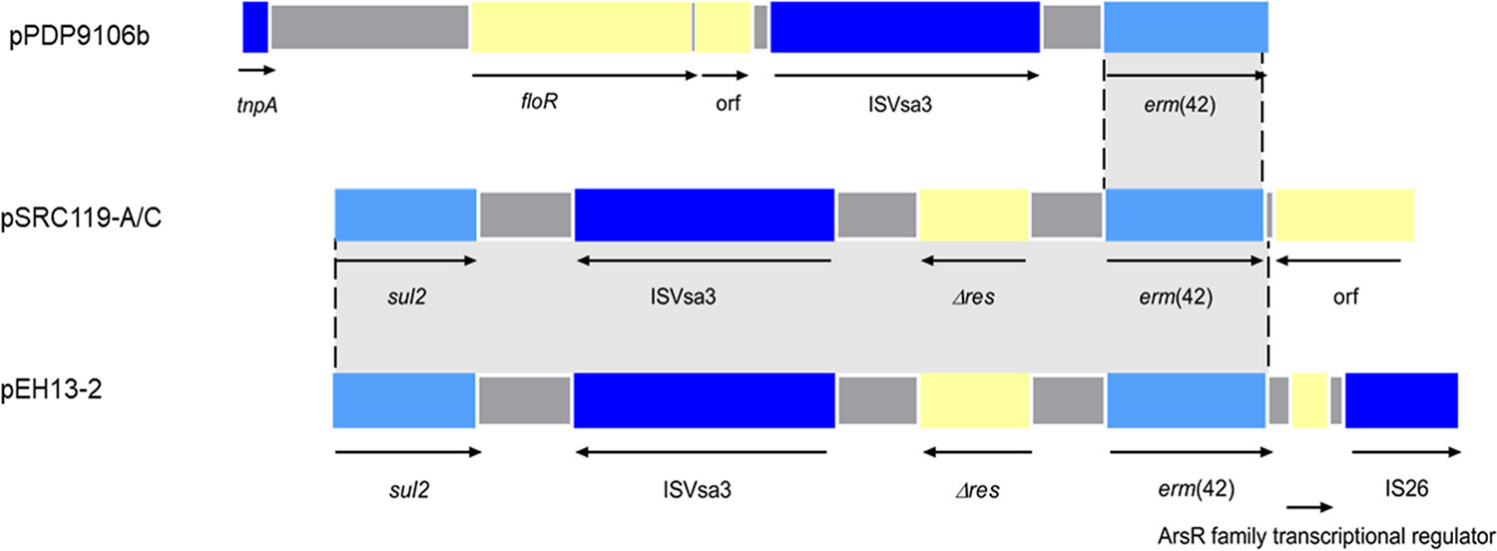
Comparison of regions surrounding *erm*(42). Structures are drawn to scale from GenBank accession numbers AB601890 (pPDP9106b) and KM670336 (pSRC119-A/C). Shared regions are indicated by shading. Arrows indicate the direction of transcription.

We then tried to identify the origin of the *erm*(42) gene in pEH13_2, as this element has not been reported in K. pneumoniae before. The *erm*(42) gene was found to be flanked by transposable elements IS*26* and IS*Vsa3* (Fig. 2b). To investigate whether the *erm*(42) gene originated from a transposon, a pair of outward-facing primers targeting the *erm*(42) gene was used to detect the potential of the neighbor nucleotides to circularize. No band was detected in gel electrophoresis of the PCR product, which suggested that the *erm*(42) gene did not originate from a transposon. BLAST results showed that the region exhibited high similarity to part of a resistance island ARI-B in type 2 A/C2 plasmid pSRC119-A/C (GenBank accession number KM670336). The pSRC119-A/C plasmid was recovered from Salmonella enterica serovars, which has been described associated with *erm*(42) gene (15). ARI-B was first described in K. pneumoniae, which contains *sul* and a variable set of additional resistance genes, while the *erm*(42) gene has been incorporated into ARI-B in pSRC119-A/C (15, 20). Only two references for the *erm*(42) gene were available in GenBank, from chromosomal DNA of Pasteurella multocida (GenBank accession number CP003022) and plasmid pPDP9106b (accession number AB601890) from Photobacterium damselae subsp. *piscicida*, in which the genetic backgrounds of *erm*(42) were highly similar. The regions surrounding *erm*(42) in pPDP9106b, pSRC119-A/C, and pEH13_2 were compared (Fig. 3). The region from *erm*(42) to *sul* in pEH13_2 was identical to that in plasmid pSRC119-A/C (Fig. 3). It might be possible that the *erm*(42) element originated from the ARI-B island in pSRC119-A/C, which would also explain why Salmonella transconjugants have greater MICs of azithromycin than the donor strain.

Azithromycin has been considered an effective antimicrobial agent for treatment of gastrointestinal and systemic infections caused by multidrug-resistant (MDR) strains of diarrheagenic Escherichia coli, *Shigella* species, and Salmonella species (13). However, selection pressure associated with the increased usage of azithromycin has led to the emergence of azithromycin-resistant isolates. Although such resistant isolates have been reported in many countries (21–23), little is known about azithromycin resistance in Salmonella. To date, there are only two reports on the mechanism of resistance to azithromycin. First, Hooda et al. reported that a mutation in the gene that encodes the RND efflux pump AcrB protein in a typhoidal Salmonella strain conferred resistance to azithromycin (23). Second, our laboratory recently revealed the role of *erm*(B) in mediating azithromycin resistance in Salmonella (12). Besides *acrB* and *erm*(B), the *mph*(A) gene was also suggested to be associated with azithromycin resistance (21), but the underlying mechanism remains to be elucidated. In this work, we confirmed that the *erm*(42) gene can confer azithromycin resistance in several *Enterobacteriaceae* strains, especially Salmonella. Of particular concern is development of resistance to azithromycin in extensively drug-resistant (XDR) Salmonella enterica subsp. *enterica* serovar Typhimurium, as azithromycin is the only remaining oral drug that can be used to effectively treat XDR typhoid infection (24). Acquisition of the plasmid that harbors the *erm*(B)/*erm*(42) gene or mutations in the *acrB* gene will apparently diminish the clinical effectiveness of azithromycin in treatment of Salmonella infections. Our findings imply that it is necessary to unveil other possible molecular mechanisms of azithromycin resistance in clinically important bacterial pathogens.

### Conclusion.

In this study, we identified a plasmid that carries three macrolide resistance genes: *erm*(B), a novel *erm*(42) gene, and *mph*(A). This plasmid, recoverable from a clinical K. pneumoniae strain, was found to encode azithromycin resistance in various bacterial species. This is the first report that *erm*(42) that can mediate azithromycin resistance in *Enterobacteriaceae*. The ability of this plasmid to be conjugated to strains of E. coli and Salmonella and encode high-level azithromycin resistance highlights a need to monitor and prevent the dissemination of this plasmid among Gram-negative bacteria, especially Salmonella.

## MATERIALS AND METHODS

### Bacterial strains, plasmids, and growth conditions.

The strains and plasmids used in this work are listed in Table 2. K. pneumoniae strain EH13 was isolated from a blood sample of an inpatient from a hospital in Hong Kong and confirmed by MALDI-TOF. E. coli strains 25922 and J53 and Salmonella enterica subsp. *enterica* serovar Typhimurium strain PY1 were recovered from our laboratory stocks. Strains were cultivated in Luria-Bertani (LB) broth medium at 37°C with shaking. Antibiotic was used at a concentration of 50 μg/mL of kanamycin.

**TABLE 2:** Strains and plasmids used in this study

Strain or plasmid	Relevant genotype	Source or reference
Strains		
E. coli		
DH5α	F^−^ ϕ80*lacZ*ΔM15 Δ(*lacZYA*-*argF*)*U169 recA1 endA1 hsd*R17(r_K_^−^ m_K_^+^) *phoA supE44 thi*-*1 gyrA96 relA1* λ^−^	Invitrogen
J53	Derivative of E. coli K-12; azide resistant	Laboratory stock
25922	Quality control strain	ATCC
K. pneumoniae EH13	Clinical strain; AZI^r^; *erm(B)*	This study
S. enterica subsp*. enterica* serovar Typhimurium PY1 (14028s)	Derivative of CDC6516-60	ATCC

Plasmids		
pCR2.1 TOPO	Amp^r^; Kan^r^; pUC ori TA cloning vector; topoisomerase I	Invitrogen
pCR2.1/*erm(42)*	*erm*(42) in pCR2.1	This study

### Cloning of the *erm*(42) gene.

The cloning of *erm*(42) gene was performed as previously described (12), using primers erm42-F (ATAGAGCGCAGGCTGAATAA) and erm42-R (AATATAGCCTGCGTCAATCG).

### Conjugation assay.

Conjugation experiments were conducted as previously described (12), using sodium azide (NaN_3_)-resistant E. coli strain J53 and S. enterica subsp. *enterica* serovar Typhimurium strain PY1 as recipients. Conjugants were confirmed by PCR targeting the *erm*(42) gene. The MIC profiles of transconjugants were also determined for the confirmation and differentiation from donor bacteria. XbaI digestion and S1 nuclease pulsed-field gel electrophoresis (PFGE) were performed to confirm successful transfer of this plasmid through conjugation.

### Antibiotic susceptibility tests.

Antimicrobial susceptibility of the test strains was determined by performing the microdilution method. The susceptibility was interpretated according to *Performance Standards for Antimicrobial Susceptibility Testing* by the Clinical and Laboratory Standards Institute (CLSI) (25). Antimicrobial agents tested included azithromycin, cefotaxime, ceftazidime, ciprofloxacin, chloramphenicol, aztreonam, ampicillin, gentamicin, amikacin, meropenem, colistin, and tigecycline. E. coli strain 25922 served as a quality control strain. All tests were performed in duplicate, and each test included three biological replicates per strain.

### DNA sequencing and bioinformatics.

DNA sequencing and bioinformatics analysis were performed as previously described (12). Genomic DNA of strain EH13 was extracted using the genomic purification kit for bacteria (Invitrogen, USA) according to the manufacturer’s guide. The extracted DNA was then subjected to library preparation with a NEBNext Ultra II DNA library preparation kit for Illumina (New England Biolabs, USA) and sequenced via the 150-bp paired-end Illumina NextSeq 500 platform (Illumina, San Diego, CA). Genomic DNA was also subjected to analysis on the long-read Oxford Nanopore Technologies MinION platform following the manufacturer’s instructions (Nanopore, Oxford, United Kingdom). Both short and long reads were *de novo* hybrid assembled using Unicycler v0.4.7 (26). Assembled genome sequences were annotated with RAST v2.0 (27). The BLAST command lines, with an 80% coverage and identity cutoff, were used to map genome sequences against the antibiotic resistance genes and plasmid replicons. The resistance genes and plasmid replicons databases were obtained from the Center for Genomic Epidemiology (http://www.genomicepidemiology.org/). Alignment of plasmid sequences with similar structures was generated by Easyfig_win_2.1 (28).

### Data availability.

Complete sequences of the chromosome of strain EH13 and plasmids pEH13_1 and pEH13_2 have been deposited in the GenBank databases under accession numbers CP089097 to CP089099.
